# Nivolumab plus cabozantinib in metastatic renal cell carcinoma: real-world evidence from the international ARON-1 study

**DOI:** 10.3389/fonc.2025.1605282

**Published:** 2025-07-25

**Authors:** Maria T. Bourlon, Luca Galli, Enrique Grande, Se Hoon Park, Bohuslav Melichar, Timothy J. Schieber, Maria José Juan-Fita, Yüksel Ürün, Javier Molina-Cerrillo, Teresa Alonso-Gordoa, Ugo De Giorgi, Jakub Kucharz, Esther Pérez Calabuig, Vincenza Conteduca, Tarek Taha, Pasquale Rescigno, Hussam Abu-Sini, Gian Paolo Spinelli, Ray Manneh Kopp, Alessia Salfi, Dipen Bhuva, Paola Valdez-Sandoval, Sofia Mendez-Bribiesca, Ondrej Fiala, Sebastiano Buti, Fernando Sabino Marques Monteiro, Aristotelis Bamias, Marwan Ghosn, Francesco Massari, Jawaher Ansari, Matteo Santoni

**Affiliations:** ^1^ Department of Hemato-Oncology, Instituto Nacional de Ciencias Medicas y Nutricion Salvador Zubiran, Mexico City, Mexico; ^2^ Escuela de Medicina, Universidad Panamericana, Mexico City, Mexico; ^3^ Oncology Unit 2, University Hospital of Pisa, Pisa, Italy; ^4^ Department of Medical Oncology, MD Anderson Cancer Center Madrid, Madrid, Spain; ^5^ Department of Hematology-Oncology, Samsung Medical Center, Sungkyunkwan University School of Medicine, Seoul, Republic of Korea; ^6^ Department of Oncology, Faculty of Medicine and Dentistry, Palacký University, Olomouc, Czechia; ^7^ Division of Medical Oncology, Department of Internal Medicine, University of Kansas Cancer Center, Westwood, KS, United States; ^8^ Department of Medical Oncology, Fundacion Instituto Valenciano de Oncologia, Valencia, Spain; ^9^ Department of Medical Oncology, Ankara University Faculty of Medicine, Ankara, Türkiye; ^10^ Department of Medical Oncology, Hospital Ramón y Cajal, Madrid, Spain; ^11^ Department of Medical Oncology, IRCCS Istituto Romagnolo per lo Studio dei Tumori (IRST) “Dino Amadori”, Meldola, Italy; ^12^ Department of Uro-Oncology, Maria Sklodowska-Curie National Research Institute of Oncology Warsaw, Warsaw, Poland; ^13^ Medical Oncology Department, CHU Insular-Materno Infantil, Las Palmas de Gran Canaria, Spain; ^14^ Unit of Medical Oncology and Biomolecular Therapy and C.R.E.A.T.E - Center for Research and Innovation Medicine, Department of Medical and Surgical Sciences, University of Foggia, Policlinico Riuniti, Foggia, Italy; ^15^ The Institute of Cancer Research, London, United Kingdom; ^16^ Royal Marsden NHS Foundation Trust, London, United Kingdom; ^17^ Translational and Clinical Research Institute, Centre for Cancer, Newcastle University, Newcastle upon Tyne, United Kingdom; ^18^ Oncology Institute, Haifa, Israel; ^19^ UOC Oncologia Territoriale Ausl Latina, Aprilia, Italy; ^20^ Clinical Oncology, Sociedad de Oncología y Hematología del Cesar, Valledupar, Colombia; ^21^ Department of Medical Oncology, Army Hospital Research and Referral, New Delhi, India; ^22^ Department of Oncology and Radiotherapeutics, Faculty of Medicine and University Hospital in Pilsen, Charles University, Pilsen, Czechia; ^23^ Biomedical Center, Faculty of Medicine in Pilsen, Charles University, Pilsen, Czechia; ^24^ Medical Oncology Unit, University Hospital of Parma, Parma, Italy; ^25^ Department of Medicine and Surgery, University of Parma, Parma, Italy; ^26^ Genitourinary Cancer Group, Latin American Cooperative Oncology Group - LACOG, Porto Alegre, Brazil; ^27^ Oncology and Hematology Department, Hospital Sírio Libanês, Brasília, Brazil; ^28^ 2nd Propaedeutic Department of Internal Medicine, ATTIKON University Hospital, School of Medicine, National and Kapodistrian University of Athens, Athens, Greece; ^29^ Hematology-Oncology Department, Faculty of Medicine, Saint Joseph University of Beirut, Beirut, Lebanon; ^30^ Medical Oncology, IRCCS Azienda Ospedaliero-Universitaria di Bologna, Bologna, Italy; ^31^ Department of Medical and Surgical Sciences (DIMEC), University of Bologna, Bologna, Italy; ^32^ Medical Oncology, Tawam Hospital, Al Ain, United Arab Emirates; ^33^ Medical Oncology Unit, Macerata Hospital, Macerata, Italy

**Keywords:** nivolumab plus cabozantinib, real-world evidence, metastatic renal cell carcinoma, clear cell renal cell carcinoma, non-clear cell renal cell carcinoma

## Abstract

**Introduction:**

Four approved immune-based combinations for untreated metastatic renal carcinoma have demonstrated survival benefits. The ARON-1 study (NCT05287464) analyzed real-world data of patients with metastatic renal cell carcinoma receiving first-line immuno-oncology combinations. This sub-analysis is focused on the nivolumab plus cabozantinib effectiveness.

**Methods:**

We conducted a retrospective study across 52 centers in 17 countries, including patients with metastatic renal carcinoma treated with first-line nivolumab plus cabozantinib, regardless of histologic characteristics, performance status, or risk by IMDC prognostic model. Patients with incomplete medical data were excluded. The primary objective of this sub-analysis of the ARON-1 study was to evaluate the real-world effectiveness and safety.

**Results:**

A total of 333 patients were treated with nivolumab plus cabozantinib, clinical characteristics included ECOG performance status ≥2 20%, non-clear cell histology 16%, sarcomatoid de-differentiation 12%, and poor-risk by IMDC 28%. At a median follow-up of 15.9 months (95%CI 11.2-44.0), the median overall survival was not reached (40.0–NR), the probability of survival at 2 years was 75%, while median progression free survival was 33.7 months (95%CI 21.1-38.9). In the entire cohort, an objective response was observed in 58%, with 6% complete responses, and a median duration of response of 38.9 months (95%CI 33.7–NR). At multivariate analysis, adverse prognostic factors for overall survival included ECOG performance status ≥2, sarcomatoid de-differentiation, brain and bone metastases, and poor IMDC group. In the safety analysis, the incidence of grade 3 or higher toxicity was 37%, with hypertension and hand-foot syndrome being the most frequent adverse events.

**Conclusion:**

The findings in the present real-world study reaffirm the clinical benefits and safety of the nivolumab plus cabozantinib combination across all subgroups, including populations that are generally excluded from clinical trials for whom data is often missing. Poor performance status, sarcomatoid de-differentiation, bone or central nervous system metastases and IMDC poor risk group were confirmed as negative prognostic factors.

## 1 Introduction

The introduction of immune-based combination therapies, with either two immune checkpoint inhibitors (ICI) or combinations of ICI with vascular endothelial growth factor receptor tyrosine kinase inhibitor (VEGFR-TKI), has dramatically changed the treatment landscape and prognosis for patients with advanced renal cell carcinoma (RCC). All these regimens, including nivolumab plus ipilimumab, pembrolizumab plus axitinib, nivolumab plus cabozantinib, and pembrolizumab plus lenvatinib, have demonstrated significant survival benefits compared to the prior standard (1–8).

The CheckMate 9ER trial demonstrated that nivolumab plus cabozantinib compared to sunitinib significantly improved the overall survival (OS) of mRCC patients by reducing the risk of death by approximately 40%; as well as improving the median progression-free survival (PFS, 16.6 *vs* 8.3 months) and the overall response rate (ORR, 55% *vs* 27%), while maintaining a manageable toxicity profile (5, 6).

Real-world data represent a way to integrate the results from phase 3 trials, focusing on subpopulations often not included in clinical trials and allowing for hypothesis generating head-to-head comparisons. It also allows clinicians to assess less frequent or late-onset toxicities.

In this context, the ARON-1 study (NCT05287464, www.aronwg.com) aimed to analyze real-world treatment outcomes for mRCC patients across multiple centers worldwide. In this report, we present a retrospective multicenter analysis of the outcomes of mRCC patients receiving first-line nivolumab plus cabozantinib in 17 countries across Europe, Asia, and North and South America.

## 2 Materials and methods

### 2.1 Study population

We conducted a retrospective data collection from patients aged 18 years or older who had a histologically confirmed diagnosis of RCC, and metastatic disease either histologically or radiologically confirmed. Data were gathered from patients with mRCC treated with first-line nivolumab plus cabozantinib between January 1, 2021, and September 1, 2024, across 52 centers in 17 countries.

Nivolumab was typically administered intravenously at a dose of 240 mg every 2 weeks or 480 mg every 4 weeks, while cabozantinib was given orally once daily, with starting doses ranging from 20 mg to 40 mg based on physician choice and patient tolerance. First-line treatment continued until there was evidence of clinical or radiological progression, intolerable toxicities, clinical judgement or patient preference or death. Imaging studies, including contrast-enhanced CT or MRI scans, were performed according to local protocols every 8 or 12 weeks. Physical exams and laboratory tests were conducted every 4 to 6 weeks throughout treatment according to local protocols.

For each patient, retrospective data were collected from patient charts (electronic or paper), including details on age, sex, histology, IMDC risk group, nephrectomy status, metastasis sites, treatment-related adverse events, oncological outcomes, and subsequent treatments after first-line therapy. Patients with insufficient data for tumor assessment or those lost to follow-up were excluded from the analysis.

### 2.2 Study endpoints

The primary objective of this sub-analysis of the ARON-1 study was to evaluate the real-world activity, effectiveness, and safety of first-line nivolumab plus cabozantinib in mRCC patients. Tumor response was assessed using RECIST 1.1 (9), categorizing outcomes as complete response (CR), partial response (PR), stable disease (SD), or progressive disease (PD). The ORR was defined as the proportion of patients achieving a CR or a PR according to RECIST 1.1 (9).

OS was defined as the time from the initiation of first-line treatment to death from any cause, while PFS was the time from the start of treatment to disease progression or death from any cause. Duration of response (DOR) was defined as the time from the start of nivolumab plus cabozantinib to progression or death in patients who achieved CR or PR, whichever occurred first.

Landmark analysis was performed designating 6 and 12 months as the time point during follow-up period to reduce potential biases related to the follow-up time.

Adverse events retrospectively collected from patient charts were graded according to the National Cancer Institute Common Terminology Criteria for Adverse Events, version 5.0. Patients without tumor progression, those who moved to subsequent lines of treatment, or those lost to follow-up at the time of analysis were censored at their last available follow-up.

### 2.3 Statistical analysis

Statistical analysis was conducted using MedCalc software (version 19.6.4, MedCalc Software, Broekstraat 52, 9030 Mariakerke, Belgium). Descriptive statistics were used to summarize the characteristics of the study population. Survival outcomes, including OS, PFS, and DOR, were analyzed using the Kaplan-Meier method, with 95% confidence intervals (95% CI) calculated according to Rothman. The median follow-up was calculated using the reverse Kaplan-Meier estimator. Differences in survival distributions were assessed using the log-rank test.

Univariate and multivariate analyses were carried out using Cox proportional hazards models, with hazard ratios (HR) and corresponding 95% CI reported. Fisher’s exact test was used for pairwise comparisons of categorical variables, and chi-square tests were applied for multiple categorical comparisons. P-values of less than 0.05 were considered statistically significant. A significance threshold of *p*<0.05 was set for all tests, and all *p*-values were two-sided.

### 2.4 Ethics approval

The ARON-1 project received approval from the Ethics Committee of the Marche Region (2021-492) and from the Institutional Review Boards of all participating centers according to the regulations in force in each Country. The study was carried out in compliance with Good Clinical Practice and international ethical standards for biomedical research. The study protocol was developed in accordance with the ethical principles outlined in the Declaration of Helsinki regarding human research.

## 3 Results

### 3.1 Patient population

Three hundred and thirty-three patients treated with first-line cabozantinib plus nivolumab were selected among the 4977 patients from the ARON-1 dataset (**Supplementary Figure S1**). The median age was 63 years (range 38–86); 241 patients (72%) were males and 92 females (28%).

In 276 patients (82%) tumor histology was clear cell RCC. In those with non-clear cell histology (57 patients), papillary histology was seen in 42 patients (13%), cromophobe in 5 patients (2%), and 10 patients had other non-clear cell histologies (3%). Sarcomatoid de-differentiation was observed in 40 patients (12%). Two hundred patients (60%) underwent nephrectomy.

Lungs (60%) and distant lymph nodes (49%) were the most common metastatic sites, with 216 patients (65%) having more than 2 affected sites. The distribution of patients according to the International mRCC Database Consortium (IMDC) criteria was good, intermediate and poor risk in 59 (18%), 180 (54%) and 94 patients (28%), respectively. **Table 1** describes the characteristics of the study population.

**Table 1:** Patient characteristics.

Characteristics	Overall no. (%)
Total patients	333 (100)
Gender	
Male Female	241 (72) 92 (28)
Age (years)	
Median (range)	63 (38–86)
ECOG-PS	
0-1 ≥2	290 (87) 43 (13)
Histologic type	
Clear cell histology Papillary Cromophobe Others	276 (82) 42 (13) 5 (2) 10 (3)
Sarcomatoid de-differentiation	
Present	40 (12)
History of surgery	
Previous nephrectomy	200 (60)
Site of metastasis	
Lung Distant lymph nodes Bone Liver Brain	200 (60) 162 (49) 132 (40) 69 (21) 17 (5)
Number of metastatic sites	
≥2 metastatic sites	216 (65)
Metastasis at diagnosis	
Yes	173 (52)
IMDC Prognostic Risk Group	
Favorable Intermediate Poor	59 (18) 180 (54) 94 (28)

### 3.2 Overall survival analysis

**Figure 1:**
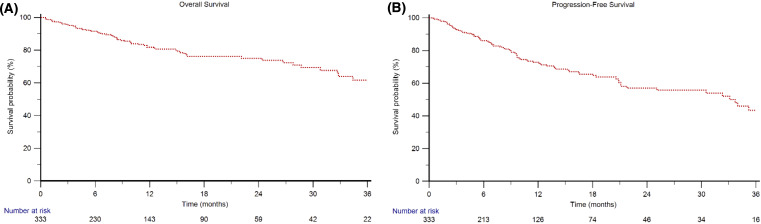
Overall Survival **(A)** and Progression-Free Survival **(B)** in mRCC patients treated with first-line nivolumab plus cabozantinib.

The median follow-up time was 15.9 months (95% CI 11.2–44.0). The median OS in the overall study population was not reached (NR, 95% CI 40.0–NR, **Figure 1**), with a 2-year OS rate of 75%. In the 6- and 12-month OS landmark analyses, the median OS was NR (**Supplementary Figure S2**).

Survival analysis was made in different subgroups. The median OS was NR in both patients aged <70 years and ≥70 years, 2-year OS rates were 80% *vs* 74% (p=0.401). The median OS was NR in both males and females (p=0.578), with a 2y-OS rate of 77% *vs* 69% (p=0.265).

**Figure 2:**
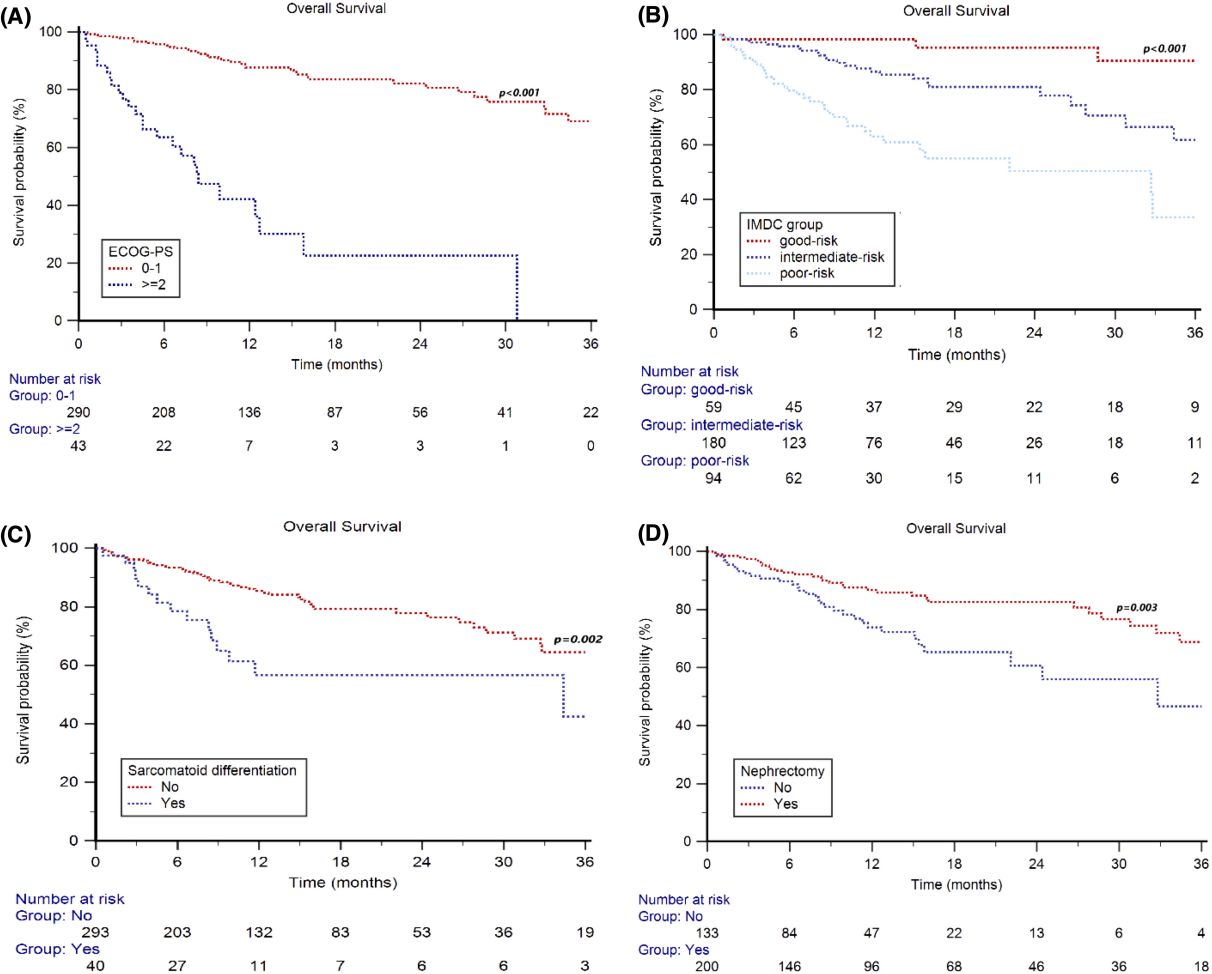
Overall Survival in mRCC patients treated with first-line nivolumab plus cabozantinib stratified by ECOG Performance Status (0–1 vs ≥2) **(A)**, IMDC group (good vs intermediate vs poor-risk) **(B)**, sarcomatoid differentiation (no vs yes) **(C)** and nephrectomy (no vs yes) **(D)**.

Median OS in the overall study population stratified by ECOG performance status (PS) was NR in patients with ECOG PS=0–1 and 8.4 months (95% CI 4.5–30.8) in patients with ECOG PS ≥2 (p<0.001, **Figure 2**), with a 2 year-OS rate of 82% *vs* 23% (p<0.001).

By stratifying patients based on IMDC group, the median OS was NR in all the three prognostic groups (p<0.001, **Figure 2**), with a 2y-OS rate of 95%, 81% and 50% (p<0.001) in good-, intermediate- and poor-risk patients, respectively.

Regarding tumor histology, the median OS was NR in patients with clear cell RCC, while it was 27.8 months (95%CI 15.4–36.5) in patients with non-clear cell histologies (p=0.784), with a 2y-OS rate of 76% *vs* 64%, respectively (p=0.089).

Furthermore, the median OS was NR in both patients with and without sarcomatoid de-differentiation (p<0.001, **Figure 2**), with a 2y-OS rate of 57% *vs* 78% (p=0.002), respectively.

The median OS was NR in patients who underwent nephrectomy and was 32.8 months (95%CI 22.1–36.9) in subjects who did not have a nephrectomy (p<0.001, **Figure 2**), with a 2 year-OS rate of 83% *vs* 61% (p<0.001), respectively.

**Figure 3:**
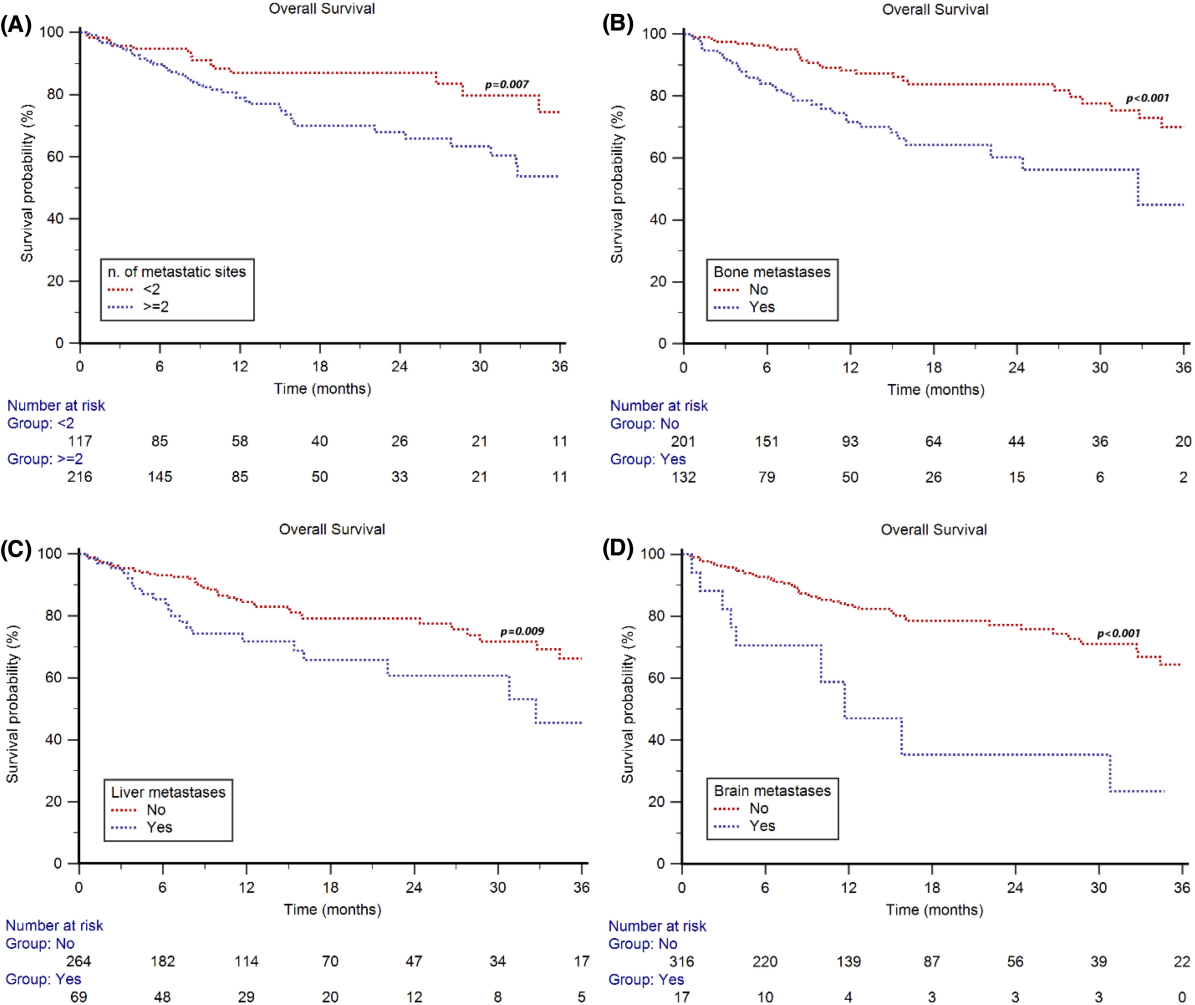
Overall Survival in mRCC patients treated with first-line cabozantinib plus nivolumab stratified by number and type of metastatic sites: number of metastatic sites (2 vs ≥2) **(A)**, bone metastasis (no vs yes) **(B)**, liver metastasis (no vs yes) **(C)**, and brain metastasis (no vs yes) **(D)**.

After stratifying the patients by metastatic sites, the median OS was NR in both patients with <2 and ≥2 metastatic sites (p=0.007, **Figure 3**), with a 2y-OS rate of 87% *vs* 68% (p=0.002). The median OS was NR in patients with or without metastases to the lungs (p=0.245, 2y-OS rate 76% *vs* 74%, p=0.747) or to distant lymph nodes (p=0.073, 2y-OS rate = 81% *vs* 69%, p=0.072), while significant differences were observed between patients with or without metastases to the bone (32.7 months, 95%CI 22.1–40.0 *vs* NR, p<0.001, **Figure 3**; 2y-OS rate = 60% *vs* 84%, p<0.001), liver (32.5 months, 95%CI 22.1–44.0 *vs* NR, p=0.009, **Figure 3**; 2y-OS rate = 60% *vs* 79%, p=0.005) or brain metastases (11.7 months, 95%CI 3.5–30.8 *vs* NR, p<0.001, **Figure 3**; 2y-OS rate = 35% *vs* 77%, p<0.001).

### 3.3 Prognostic factors in patients receiving first-line nivolumab plus cabozantinib

In the whole study population, ECOG-PS, nephrectomy, sarcomatoid de-differentiation, IMDC group, bone, liver and brain metastases were significantly associated with OS at univariate analysis (**Table 2**). At multivariate analysis, ECOG-PS ≥2, sarcomatoid de-differentiation, IMDC poor risk group, bone or brain metastases were significantly associated with shorter OS (**Table 2**).

**Table 2:** Univariate and multivariate analysis in mRCC patients receiving first-line nivolumab plus cabozantinib.

Overall Survival (Overall population)	Univariate Cox Regression		Multivariate Cox Regression	
	HR (95%CI)	*p-value*	HR (95%CI)	*p-value*
Sex (females vs males)	1.16 (0.68−1.98)	0.578		
Age (>=70y vs <70y)	1.02 (0.59−1.75)	0.950		
Geographical area (Rest of the world vs United States or Europe	1.32 (1.01−1.72)	**0.040**	1.29 (1.02-1.63)	**0.036**
ECOG-PS ≥2 (yes vs no)	8.59 (5.07−14.56)	**<0.001**	4.38 (2.46-7.79)	**<0.001**
Nephrectomy (yes vs no)	0.49 (0.30−0.80)	**0.004**	0.90 (0.52−1.54)	0.698
Histology (nccRCC vs ccRCC)	1.10 (0.54−2.26)	0.784		
Sarcomatoid differentiation (yes vs no)	2.56 (1.45−4.50)	**0.001**	2.38 (1.32−4.31)	**0.004**
IMDC group (poor vs intermediate/good)	3.09 (2.08−4.60)	**<0.001**	1.92 (1.22−3.02)	**0.005**
Lung metastases (yes vs no)	0.75 (0.45−1.22)	0.247		
Distant lymph node metastases (yes vs no)	1.56 (0.96−2.54)	0.076		
Bone metastases (yes vs no)	2.81 (1.70-4.65)	**<0.001**	1.98 (1.14-3.43)	**0.014**
Liver metastases (yes vs no)	1.95 (1.17−3.25)	**0.010**	1.50 (0.89−2.54)	0.130
Brain metastases (yes vs no)	3.63 (1.79−7.36)	**<0.001**	2.37 (1.14-4.96)	**0.022**

ccRCC = clear cell Renal Cell Carcinoma; IMDC = International Metastatic RCC Database Consortium; nccRCC = non-clear cell Renal Cell Carcinoma.Bold values indicate results that reached statistical significance.

### 3.4 Response to first-line nivolumab plus cabozantinib

In the whole study population, 19 CR (6%), 174 PR (52%), 103 SD (31%) and 37 PD (11%) were observed, with an ORR of 58%. The 2y-OS rates were 100%, 87%, 65% and 24% in patients with CR, PR, SD or PD, respectively (*p*<0.001).

The median DOR was 38.9 months (95%CI 33.7–NR) for the 58% patients who had an ORR. In patients who achieved CR the DOR was NR, and in those who had a PR the DOR was 35.2 months (95%CI 32.3–44.0).

By stratifying patients based on IMDC groups, in the good-risk group we observed 12 CR (20%), 27 PR (46%), 16 SD (29%) and 4 PD (5%), with an ORR of 66%; in the intermediate-risk group, 6 CR (4%), 97 PR (54%), 64 SD (35%) and 13 PD (7%) were demonstrated, with an ORR of 58%; and in the poor-risk group, 1 CR (1%), 50 PR (53%), 22 SD (24%) and 21 PD (18%) were found, with an ORR of 54%.

In the 276 patients with clear cell RCC, we reported 16 CR (7%), 148 PR (53%), 79 SD (28%) and 33 PD (12%), with an ORR of 60%. In the 57 patients with non-clear cell histology, we reported 1 CR (2%), 30 PR (47%), 24 SD (42%) and 2 PD (9%), with an ORR of 49%.

### 3.5 Progression free survival and subsequent therapies

The median PFS was 33.7 months (95%CI 21.1–38.9, **Figure 1**). For the 6- and 12-month PFS landmark analyses, the median PFS was 38.6 months (95%CI 32.3−50.7) and 41.4 months (95%CI 34.0−51.5), respectively (**Supplementary Figure S2**). One hundred and one patients (30%) progressed during nivolumab plus cabozantinib therapy; of these, 57 (56%) received subsequent treatments (26 patients received sunitinib, 8 axitinib, 5 pazopanib, 5 pembrolizumab plus lenvatinib, 5 lenvatinib plus everolimus, 4 everolimus, 3 tivozanib and 1 was enrolled in a clinical trial). The median PFS of second-line therapy was 5.3 months (95%CI 3.3–7.2).

### 3.6 Safety, dose reduction and therapy interruptions

Grade 3-Grade 4 (G3-G4) adverse events (AEs) were observed in 122 patients (37%). Hypertension (9%), and hand-foot syndrome (8%) were the two most frequent SAEs (**Table 3**).

**Table 3:** Grade 3-Grade 4 (G3-G4) adverse events, drug interruptions and dose reductions.

Characteristics	Overall no. (%)
G3-G4 adverse events	122 (37)
G3-G4 Hypertension	29 (9)
G3-G4 Fatigue	23 (7)
G3-G4 Diarrhea	24 (7)
G3-G4 Hand-Foot Syndrome	27 (8)
G3-G4 Hypothyroidism	15 (4)
Initial cabozantinib dose reduction	7 (2)
Subsequent cabozantinib dose reductions	101 (30)
Cabozantinib interruptions due to G3-G4 adverse events	73 (22)
Nivolumab interruptions due to G3-G4 adverse events	40 (12)

Cabozantinib dose reductions were registered in 108 patients (32%), 2% started cabozantinib at 20 mg/d, 30% reduced the dose during treatment (**Table 3**); 22% and 12% of patients interrupted cabozantinib or nivolumab due to G3-G4 AEs, respectively (**Table 3**).

The median OS was NR in both patients who reduced cabozantinib dose compared to those treated with standard dose (*p*=0.322), with a 2y-OS rate of 79% *vs* 72% (*p*=0.324). Furthermore, the median OS was NR in patients who interrupted or not cabozantinib (p=0.807) or nivolumab (*p*=0.235) due to G3-G4 adverse events, with a 2y-OS rate of 80% *vs* 74% (*p*=0.401) and 83% *vs* 74% (*p*=0.168), respectively.

## 4 Discussion

The current standard of care for the first-line treatment of clear cell RCC is a combination therapy of either ICI/ICI or ICI/VEGFR-TKI, with a proven benefit in OS over the long-standing standard of care, sunitinib, in phase 3 randomized trials (1–8). The nivolumab plus cabozantinib combination has reported improved OS with a 55-month median follow-up (10).

On the other hand, given the lower frequency of non-clear cell RCC, treatment decisions in this subgroup are based on smaller phase 2 studies (11). Recently, in these histologic subtypes the combination therapies with a multi-kinase inhibitor and an ICI, including cabozantinib plus nivolumab and pembrolizumab plus lenvatinib, have proven to be effective (12, 13). The nivolumab plus cabozantinib regimen is recommended given the outcomes seen in the phase 2 trial (cohort 1) with ORR of 48%, a median PFS and median OS of 13 and 28 months, respectively (12).

Nevertheless, it is known that, more often than not, the participants in prospective clinical trials constitute a rather selected population. The importance of having real-world data is that these reports encompass populations of patients often excluded from clinical trials, such as patients with poor PS or aggressive disease, especially those with central nervous system involvement. The present study reports data of 333 patients with RCC treated with nivolumab plus cabozantinib combination in the first-line setting. To the best of our knowledge, this is the largest retrospective multicenter study exploring this therapeutic strategy to date (**Table 4**) (14–16). Furthermore, our study gathers data from 52 centers across 17 countries, offering valuable insights from a global oncology perspective by providing information across different healthcare systems, ethnic backgrounds and diverse resource settings.

**Table 4:** Real-world data studies with nivolumab plus cabozantinib.

Study	N (patients)	Findings
Oka Y, et al. (14) Retrospective	NA	Pharmacovigilance study • Worst AEs with N/C: general disorders and administration site conditions, gastrointestinal disorders, metabolism and nutrition disorders, nervous system disorders, skin and subcutaneous tissue disorders, vascular disorders
Hilser T, et al. (15) Retrospective	96	• Median PFS: 18.6 months, median OS: not reached in overall population • DCR: 76.1% • AEs: 82.3% all grades (elevated liver enzymes 34%, diarrhea 31%, hand-foot syndrome 29%)
Ku C-H., et al. (16) Retrospective	21	• Median PFS and OS: not reached • ORR 52.2%, DCR 94.7% • AEs: hand-foot syndrome, fatigue and diarrhea

PFS, progression free survival; OS, overall survival; ORR, objective response rate; DCR, disease control rate; AEs, adverse events; NA, not applied; N/C, nivolumab plus cabozantinib.

The median follow-up in the present study was 15.9 months, which is similar to the first report of the Checkmate 9ER study (18.1 months) (5). In this real-life evidence, the median OS has not been reached and the 75% OS at 2 years is close to the one reported in the phase 3 study. OS benefits were consistent irrespective of age and sex. Patients with poor PS experienced a shorter OS, and this is informative for the everyday decision making, since this subset of more frail patients is not represented in large phase 3 trials and is frequently seen in clinical grounds. As expected from previous reports, apart from poor PS, adverse prognostic factors included sarcomatoid de-differentiation, bone or brain metastases, and IMDC poor risk group (17).

Notably, median PFS and median DOR were 33.7 and 38.9 months respectively, which is higher than the rate reported for the CheckMate 9ER trial (16.6 and 20.2 months) (5, 6). Several factors may contribute to this discrepancy. Assessments in the clinical trial initially occurred every 6 weeks, while in this real-life report they occurred every 8 to 12 weeks and this could impact the PFS and DOR calculation. However, the benefit of more frequent imaging in real-world settings is debatable, as it may increase healthcare costs and patient burden without clear added value. Complications from repeated assessments, as well as their impact on quality of life, should be carefully weighed when determining imaging intervals.

Additionally, RECIST calculation in the real-world setting might not be as strict as in clinical trials and evaluation is not subjected to a central radiologic review, potentially affecting consistency and objectivity in response evaluation.

Another important consideration is the real-world practice of continuing treatment beyond radiographic progression, a strategy often restricted in clinical trials. Patients may remain on therapy due to perceived clinical benefit or personal preference, and in such cases, the date of treatment change rather than actual progression may be recorded, artificially prolonging PFS.

Regarding response rates compared to the subset of patients with clear cell RCC in the ARON-1 study, patients in the CheckMate 9ER had higher CR rates (13.5% *vs* 7%) and lower PD (6.5% *vs* 12%). These differences may be attributed to differences in patient populations. The ARON-1 trial included 43 patients (13%) with ECOG PS ≥2 and 17 patients (5%) with brain metastases, who were excluded from CheckMate 9ER, and a higher proportion of patients with poor-risk IMDC classification (28% *vs* 19% in CheckMate 9ER). Despite differences in CR and PD, ORR were similar in both trials (60% in ARON-1 *vs* 56% in CheckMate 9ER) (5, 6).

For non-clear cell RCC, the response rates in the ARON-1 study were consistent with those reported for the cohort 1 of the phase 2 trial, with an ORR of 49% and 48%, respectively. Notably, one patient in the ARON-1 trial achieved CR compared to none in the phase 2 trial. However, the rate of PD was higher in ARON-1 (9% *vs*. 3% in the phase 2 trial) (11). We consider that having real-world data in this subgroup is particularly meaningful given the difficulties to accrue patients with this histologic subtype in clinical trials.

Analyzing the safety profile, grade 3 or more toxicity was reported in 75.3% of patients with the combination in the CheckMate 9ER (5). A lower incidence of grade 3 or more toxicity was reported (37%) in the ARON-1. This could be explained in part because some toxicities such as mucositis, stomatitis, hematological disorders, dysgeusia, and liver enzyme increase were not systematically reported. In addition, in daily clinical practice, adverse events monitoring is not as rigorous as in clinical trials. Interestingly, the most frequent severe toxicities in ARON-1 were hypertension (9%), hand-foot syndrome (8%), diarrhea (7%), fatigue (7%), and hypothyroidism (4%). These frequencies are largely comparable to those reported in CheckMate 9ER, where grade ≥3 hypertension occurred in 12.5%, hand-foot syndrome in 7.5%, diarrhea in 7%, fatigue in 3.4%, and hypothyroidism in 0.3% (5). Furthermore, cabozantinib dose reductions were less frequent in ARON-1 (32%) compared to CheckMate 9ER (56%) (5), aligning with the lower incidence of grade 3 or higher toxicity and suggesting better overall treatment tolerance. Importantly, having real-world data on dose reductions and treatment discontinuations is essential, as these factors may influence treatment efficacy and patient outcomes in clinical practice.

A previous report of ARON-1 trial, which included 729 patients treated with all four approved immune-based combinations, suggests consistent efficacy results compared to the phase 3 trials in the overall population (median OS 36.5 months, median PFS 15 months, and ORR 49%) (18). Real-world data exists for all available immune-based combinations. For nivolumab and ipilimumab, real-world studies report ORR ranging from 33% to 48%, median PFS between 9 and 18 months, and median OS of 49 months—results consistent with those reported in the CheckMate 214 trial (ORR 42%, median PFS 8 months, and median OS 47 months) (1, 2, 19–21). Real-world evidence for pembrolizumab and lenvatinib is emerging, with two studies (n = 50 and n = 54) reporting ORR of 66% and 38%, respectively, the former ORR aligns with the CLEAR trial results (ORR 71%). Neither study has yet reported survival outcomes (19, 22, 23). Similarly, real-world data for pembrolizumab and axitinib indicate efficacy consistent with KEYNOTE 426, with ORR ranging from 48% to 71% compared to 60% in the trial (3, 24–26).

Specifically for the combination of nivolumab plus cabozantinib, recent real-world studies further support our findings. The GUARDIANS multicenter retrospective study, which included 96 patients, reported an ORR of 45.8% and a median PFS of 18.6 months. Median OS was not reached, with a 12-month probability of OS of 77.5%. Grade ≥3 treatment-related adverse events occurred in 41.7% of patients, and 25% discontinued treatment due to toxicity (15). Similarly, a retrospective study conducted across eight German cancer centers included 67 patients and, at a median follow-up of 8.3 months, reported an ORR of 46.3%, a 6-month PFS rate of 81.9%, and grade ≥3 AEs in 47.8% of patients (16). In another study from Taiwan by Ku et al., which included 21 patients treated with nivolumab plus cabozantinib, the ORR was 52.2% (27). These findings are consistent with the efficacy outcomes observed in ARON-1 and further reinforce the reproducibility of this combination in routine clinical practice.

We consider that ARON-1 study has several strengths, such as being a multicenter effort with global representation, it has the largest number of patients, and the inclusion of patients with characteristics (e.g. histological, performance status, brain metastasis) that are generally excluded from randomized clinical trials. However, as a retrospective study, the main limitations are a selection bias of cases included (since patients with incomplete data were excluded from the study) and the confounder variables that could not be measured. Another, limitation is the short time of follow-up compared to the phase 3 CheckMate 9ER study that has reported outcomes with 55 months median follow-up. However, the ARON-1 data will continue to mature over the years and will be informative of long-term outcomes as well.

As in most daily clinical practices, formal questionnaires or scales for evaluating quality of life are scarcely employed (28). A weakness of the real-world data from the different combinations in this scenario, including the ARON-1 trial, is the lack of information regarding how patients experience both treatment benefits and adverse events.

In conclusion, decision-making regarding the optimal immune-based combination is inherently complex. The ARON-1 trial seeks to simplify this process by providing real-world data on the combination of nivolumab and cabozantinib. The findings of this study show benefit across all subgroups, including populations that are generally excluded from clinical trials, for whom data if often missing.

## Data Availability

The raw data supporting the conclusions of this article will be made available by the authors, without undue reservation.

## References

[1] Motzer RJ Tannir NM McDermott DF Arén Frontera O Melichar B Choueiri TK. Nivolumab plus Ipilimumab versus Sunitinib in Advanced Renal-Cell Carcinoma. N Engl J Med. (2018) 378:1277–90. doi: 10.1056/NEJMoa1712126, PMID: 29562145 PMC5972549

[2] Tannir NM Albigès L McDermott DF Burotto M Choueiri TK Hammers HJ. Nivolumab plus ipilimumab versus sunitinib for first-line treatment of advanced renal cell carcinoma: extended 8-year follow-up results of efficacy and safety from the phase III CheckMate 214 trial. Ann Oncol. (2024) 35:1026–38. doi: 10.1016/j.annonc.2024.07.727, PMID: 39098455 PMC11907766

[3] Rini BI Plimack ER Stus V Gafanov R Hawkins R Nosov D. Pembrolizumab plus Axitinib versus Sunitinib for Advanced Renal-Cell Carcinoma. N Engl J Med. (2019) 380:1116–27. doi: 10.1056/NEJMoa1816714, PMID: 30779529

[4] Powles T Plimack ER Soulières D Waddell T Stus V Gafanov R. Pembrolizumab plus axitinib versus sunitinib monotherapy as first-line treatment of advanced renal cell carcinoma (KEYNOTE-426): extended follow-up from a randomised, open-label, phase 3 trial. Lancet Oncol. (2020) 21:1563–73. doi: 10.1016/S1470-2045(20)30436-8, PMID: 33284113

[5] Choueiri TK Powles T Burotto M Escudier B Bourlon MT Zurawski B. Nivolumab plus Cabozantinib versus Sunitinib for Advanced Renal-Cell Carcinoma. N Engl J Med. (2021) 384:829–41. doi: 10.1056/NEJMoa2026982, PMID: 33657295 PMC8436591

[6] Powles T Burotto M Escudier B Apolo AB Bourlon MT Shah AY. Nivolumab plus cabozantinib versus sunitinib for first-line treatment of advanced renal cell carcinoma: extended follow-up from the phase III randomised CheckMate 9ER trial. ESMO Open. (2024) 9:102994. doi: 10.1016/j.esmoop.2024.102994, PMID: 38642472 PMC11046044

[7] Motzer R Alekseev B Rha SY Porta C Eto M Powles T. Lenvatinib plus pembrolizumab or everolimus for advanced renal cell carcinoma. N Engl J Med. (2021) 384:1289–300. doi: 10.1056/NEJMoa2035716, PMID: 33616314

[8] Motzer RJ Porta C Eto M Powles T Grünwald V Hutson TE. Lenvatinib plus pembrolizumab versus sunitinib in first-line treatment of advanced renal cell carcinoma: final prespecified overall survival analysis of CLEAR, a phase III study. J Clin Oncol. (2024) 42:1222–8. doi: 10.1200/JCO.23.01569, PMID: 38227898 PMC11095851

[9] Eisenhauer EA Therasse P Bogaerts J Schwartz LH Sargent D Ford R. New response evaluation criteria in solid tumours: revised RECIST guideline (version 1.1). Eur J Cancer. (2009) 45:228–47. doi: 10.1016/j.ejca.2008.10.026, PMID: 19097774

[10] Bourlon MT. Nivolumab plus Cabozantinib *vs* Sunitinib for Previously Untreated Advanced RCC: Results from 55-Month Follow-up of the CheckMate 9ER Trial. Oral presentation. ASCO GU; 2024 Jan 25-27. (2024) San Francisco, CA, USA.

[11] Naik P Dudipala H Chen Y-W Rose B Bagrodia A McKay RR. The incidence, pathogenesis, and management of non-clear cell renal cell carcinoma. Ther Adv Urol. (2024) 12:17562872241232578. doi: 10.1177/17562872241232578, PMID: 38434237 PMC10906063

[12] Lee C-H Voss MH Carlo MI Chen Y-B Zucker M Knezevic A. Phase II trial of cabozantinib plus nivolumab in patients with non–clear-cell renal cell carcinoma and genomic correlates. J Clin Oncol. (2022) 40:2333–41. doi: 10.1200/JCO.21.01944, PMID: 35298296 PMC9287282

[13] Albiges L Gurney H Atduev V Suarez C Climent MA Pook D. Pembrolizumab plus lenvatinib as first-line therapy for advanced non-clear-cell renal cell carcinoma (KEYNOTE-B61): a single-arm, multicentre, phase 2 trial. Lancet Oncol. (2023) 24:881–91. doi: 10.1016/S1470-2045(23)00276-0, PMID: 37451291

[14] Oka Y Matsumoto J Takeda T Iwata N Niimura T Ozaki AF. Adverse events of nivolumab plus ipilimumab versus nivolumab plus cabozantinib: a real-world pharmacovigilance study. Int J Clin Pharm. (2024) 46:745–50. doi: 10.1007/s11096-024-01713-1, PMID: 38632203

[15] Hilser T Darr C Niegisch G Schnabel MJ Foller S Häuser L. Cabozantinib plus nivolumab in adult patients with advanced or metastatic renal cell carcinoma: A retrospective, non-interventional study in a real-world cohort/GUARDIANS project. Cancers (Basel). (2024) 16:2998. doi: 10.3390/cancers16172998, PMID: 39272856 PMC11393955

[16] Ku C-H Su PJ Huang WK Kuo YC Chang CF Yu S. Abstract 303P. Cabozantinib versus cabozantinib plus nivolumab in first-line treatment of advanced renal cell carcinoma: A Chang Gung medical foundation multicentric cohort, real-world study. Ann Oncol. (2024) 35:S1521. doi: 10.1016/j.annonc.2024.10.322

[17] Heng DY Xie W Regan MM Warren MA Golshayan AR Sahi C. Prognostic factors for overall survival in patients with metastatic renal cell carcinoma treated with vascular endothelial growth factor-targeted agents: results from a large, multicenter study. J Clin Oncol. (2009) 27:5794–9. doi: 10.1200/JCO.2008.21.4809, PMID: 19826129

[18] Santoni M Roviello G Grande E De Giorgi U Fiala O Seront E. Global real-world outcomes of patients receiving immuno-oncology combinations for advanced renal cell carcinoma: the ARON-1 study. Target Oncol. (2023) 18:559–70. doi: 10.1007/s11523-023-00978-2, PMID: 37369815

[19] Kojima T Kato R Sazuka T Yamamoto H Fukuda S Yamana K. Real-world effectiveness of nivolumab plus ipilimumab and second-line therapy in Japanese untreated patients with metastatic renal cell carcinoma: 2-year analysis from a multicenter retrospective clinical study (J-cardinal study). Japanese J Clin Oncol. (2022) 52:1345–52. doi: 10.1093/jjco/hyac124, PMID: 35920793 PMC9631464

[20] Ishihara H Yuki N Ishiyama R Ikeda T Kobari Y Fukuda H. Real-world outcomes of nivolumab plus ipilimumab combination therapy for advanced renal cell carcinoma in Japanese patients: data with a minimum of 3 years of follow-up. Japan Journ Clin Oncol. (2024) 54:577–83. doi: 10.1093/jjco/hyae001, PMID: 38251783

[21] Meerveld-Eggink A Graafland N Wilgenhof S Van Thienen JV Lalezari F Grant M. Primary renal tumour response in patients treated with nivolumab and ipilimumab for metastatic renal cell carcinoma: real-world data assessment. Europ Urol. (2022) 35:54–8. doi: 10.1016/j.euros.2021.11.003, PMID: 35024632 PMC8738899

[22] Hara T Suzuki K Okamura Y Chiba K Sato R Matsushita Y. Efficacy and safety of lenvatinib and pembrolizumab as first-line treatment for advanced renal cell carcinoma patients: real-world experience in Japan. Int J Clin Oncol. (2024) 29:1931–6. doi: 10.1007/s10147-024-02633-w, PMID: 39472358

[23] Stativko O Pokataev I Fedyanin M Kravchuk DA Andreiashkina I Polshina N. Real-world efficacy of lenvatinib and pembrolizumab as first-line therapy in patients with metastatic renal cell carcinoma with high burden disease. Ann Oncol. (2024) 35:S1505–30. doi: 10.1016/j.annonc.2024.10.304

[24] Shah NJ Sura SD Shinde R Shi J Singhal P Perini RF. Real-world clinical outcomes of patients with metastatic renal cell carcinoma receiving pembrolizumab + axitinib vs ipilimumab + nivolumab. Urol Oncol. (2023) 41:459.e1–8. doi: 10.1016/j.urolonc.2023.08.009, PMID: 37722984

[25] Guida A Gili A Mosillo C Maruzzo M Lai E Pierantoni F. Efficacy and Safety of Pembrolizumab plus Axitinib combination for Metastatic Renal Cell Carcinoma in a Real-World Scenario: Data From the Prospective ProPAXI Study. Clin Genitourin Cancer. (2024) 22:102225. doi: 10.1016/j.clgc.2024.102225, PMID: 39405768

[26] Zakharia Y Thomaidou D Li B Siu G Levin R Vlahiotis A. Real-world therapy management and outcomes of first-line axitinib plus pembrolizumab in patients with advanced renal cell carcinoma in the United States. Front Oncol. (2022) 12:861189. doi: 10.3389/fonc.2022.861189, PMID: 35664758 PMC9161634

[27] Ku WC Wang YT Lin CC Hsieh MC Lee CH. Real-world experience with nivolumab and cabozantinib for metastatic renal cell carcinoma in Taiwan. J Clin Oncol. (2023) 41:628. doi: 10.1200/JCO.2023.41.6_suppl.628

[28] Carr AJ Higginson IJ. Using quality of life measures in the clinical setting. BMJ. (2001) 322:1297–300. doi: 10.1136/bmj.322.7297.1297, PMID: 11375237 PMC1120388

