# Supplementary material for: Nivolumab plus cabozantinib in metastatic renal cell carcinoma: real-world evidence from the international ARON-1 study
Source: Front Oncol. 2025 Jul 25;15:1605282. doi: 10.3389/fonc.2025.1605282 (PMC12331508; doi:10.3389/fonc.2025.1605282)
Supplement: Supplementary file 1 [file DataSheet1.docx]

Supplementary Material

# Supplementary Data

## Supplementary Figures

**Figure S1.** Selection process from the ARON-1 dataset.


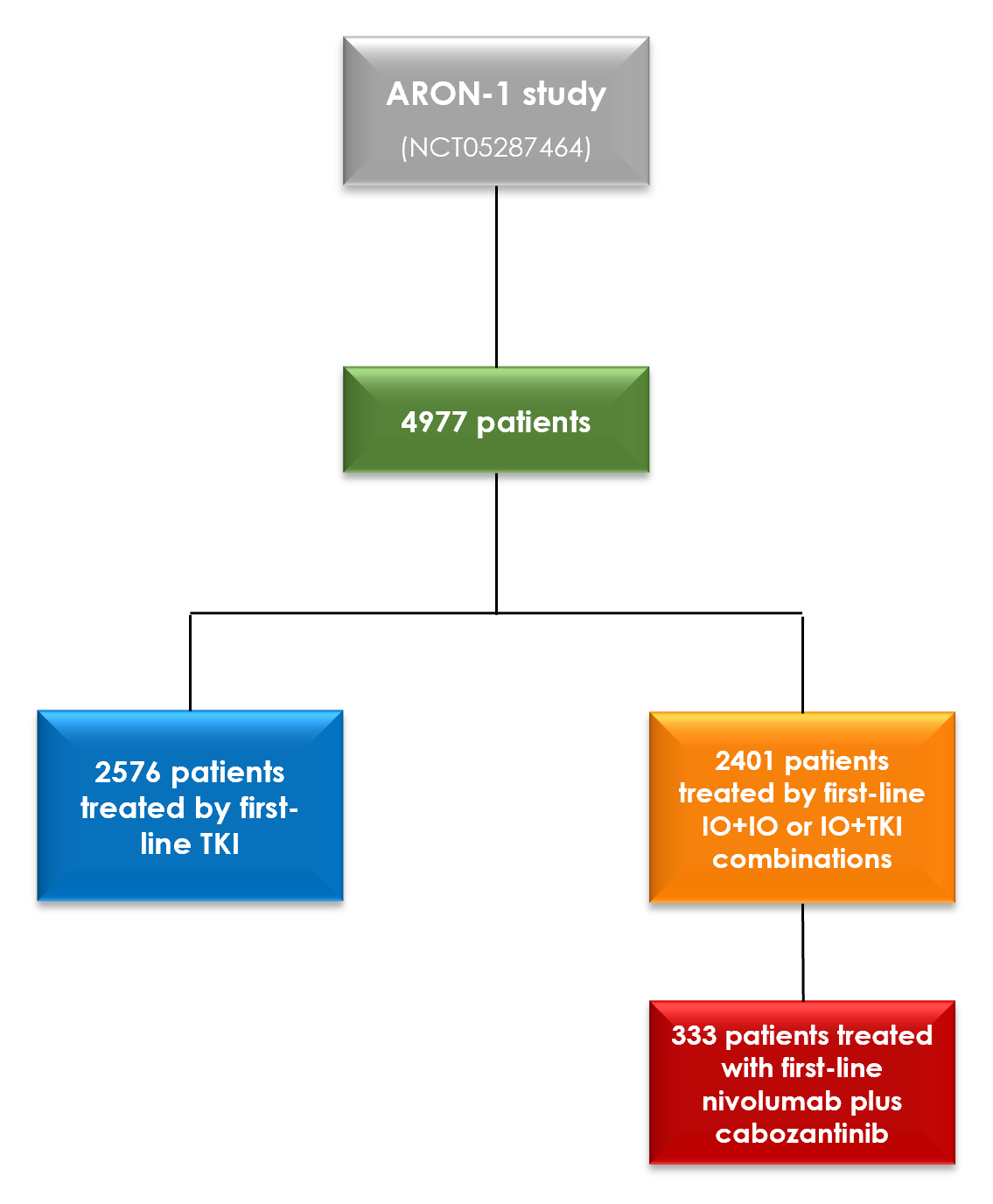


**Figure S2.** 6-months and 12-month landmark analyses for Overall Survival and Progression-Free Survival in mRCC patients treated with first-line cabozantinib plus nivolumab. **(A)** 6-month landmark overall survival, **(B)** 12-month landmark overall survival, **(C)** 6-month landmark progression-free survival, and **(D)** 12-month landmark progression-free survival.

**
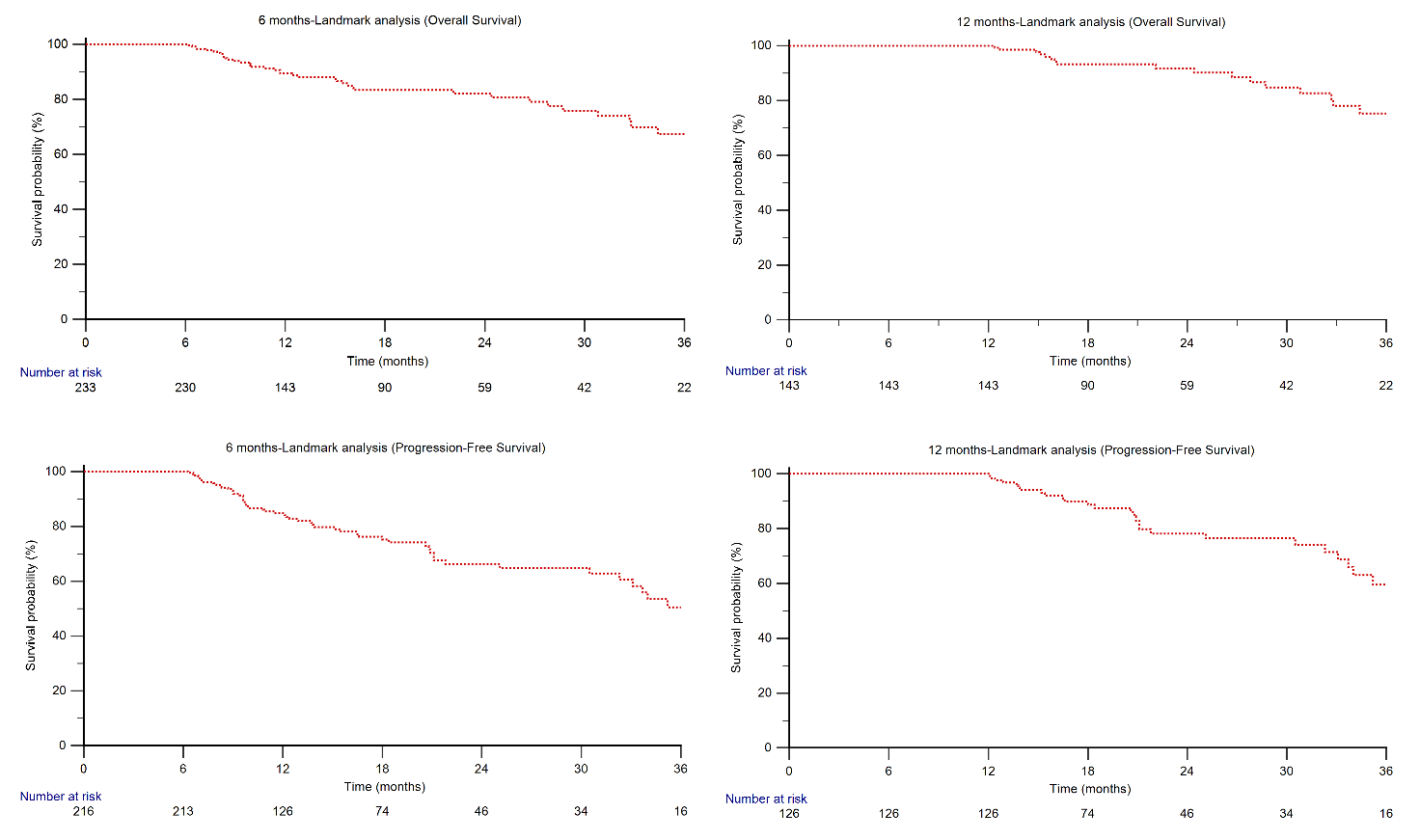
**

**(B)**

**(C)**

**(A)**

**(D)**

**
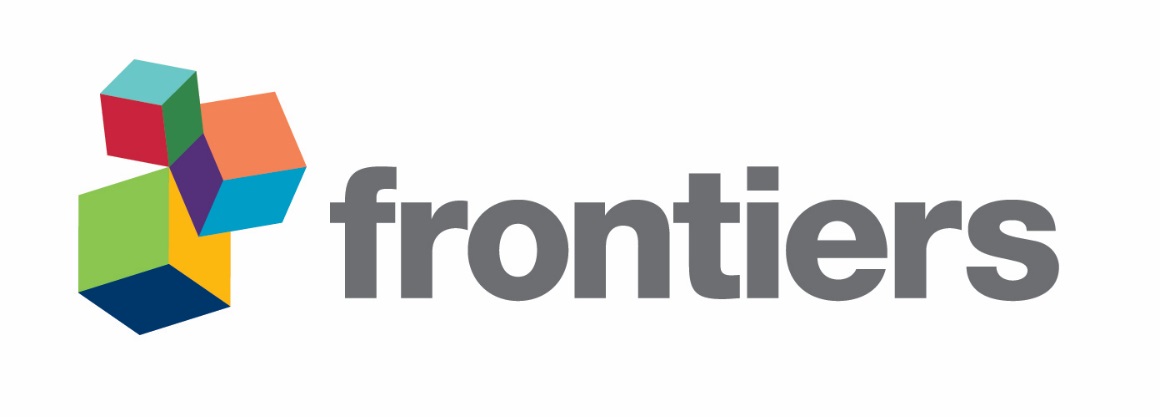
**

**Supplementary Figure 1.** The figure legends are required to have the same font as the main text, 12 point normal Times New Roman, single spaced. Please use a single paragraph for each legend and prepare the figures keeping in mind the PDF layout.
